# Furrowed Enamel in Connection with Syphilitic Diseases

**Published:** 1873-07

**Authors:** S. P. Cutler

**Affiliations:** Memphis, Tennessee.


### SELECTED ARTICLES.

### ARTICLE IV.

### *Furrowed Enamel in Connection with Syphilitic and other Exanthematous Diseases.*—Concluded.

BY S. P. CUTLER, M. D., MEMPHIS, TENNESSEE.

#### INFECTION.

Dr. Wm. W. Cable, of Pittsburgh, (*Medical and Surgical Reporter*,) speaks of communicability of both primary and secondary syphilis, by contact with mucous membranes, both primary and secondary sores ; by kissing, when the lips are affected with secondary sores, communicating the disease in its secondary form, without any of the acute symptoms, excoriated tonsils and blotches on the skin being the leading features of the reported cases.

The cases cited not only show that the disease can be conveyed from secondary sores to an untainted person, but that the person becoming so affected is capable, also, of re-communicating the disease to a third party.

The question here is, can the secondary form be produced by inoculation into the skin ? Would it have to take on the acute form ? Whether or not the duration of the acute disease would be shorter than when communicated from fresh chancres ?

The writer speaks of its communicability to the lower animals, and of its communicative character.

This horrible disease, in many of its phases, resembles small pox and its kindred types ; others again, very unlike. Small-pox may be produced by inoculation, or inhalation, though the former is less violent. Syphilis only by inoculation, and hereditary transmission, the latter never occurring in small-pox and kindred diseases, as already stated. Small-pox has its three characteristic stages, so has syphilis, though somewhat dissimilar. There is the initiatory stage, incubative stage, the febrile, or stage of systemic reaction, then subsiding into the third stage, in the one case, convalescence, in the other, constitutional taint, fixed in the organism, and transmissible: In the one case, immunity from genuine subsequent attacks, as a rule; in the other, not, as a rule, only as an exception; the rule is a modified form, somewhat as in small-pox.

I am not quite certain that it is entirely safe for a healthy person to inhale the breath, for a considerable length of time, of a person, during the febrile stage of syphilis. If no actual taint is transmitted, there might be a very unhealthy thy condition of the blood established in certain susceptible subjects, that might degenerate into some form of constitutional disease, of a non-infection or contagious nature. I do not claim that such actually takes place often, if at all.

### ANOTHER CASE OF FURROWED ENAMEL.

I saw a young man, a short time since, about 25 years old, of sanguine temperament, middle size, hair light, eyes blue, form good, whose twelve front teeth had two deep grooves across the enamel, on the labial surface, near their cutting edges, the ends contracted and notched, though sound and firm. Two of the lower molars, on one side, were decayed, one a bad, the other a small decay; all the others were sound. There was a peculiar bluish tint of the gums and other soft parts of the mouth; the breath bad, and indicative of some constitutional disease or disturbance, which is unusual in healthy persons. I made inquiry as to whether he had had measles, scarlatina, or any other cutaneous disease, when a child? to which he answered in the negative. He had a peculiar look about his face that I would pronounce, *after seeing his teeth*, to be the result of hereditary syphilis, or a syphilitic taint inherited. The blood, under the microscope, gave no unusual or abnormal appearances whatever.

This man said he was stout and healthy, which leads us to conclude that he did not suffer from the taint; the appearances of the blood under the microscope would lead to the same conclusion; the bad breath being the main indication of any constitutional disturbance at present.

It will be neccessary to assign some reason for this defective enamel development. My views are these:

In the formation of the teeth, there must be a double basement, or formative membrane, situated between the dentine and enamel, at the commencement of the formative process, one process traveling inwards, the other outwards, both commencing about the same time, by consecutive improvised primordial ossifying cells, or cells that are to undergo ossification. In the enamel, all cells ossify. On the contrary, in the dentinal cells, there are two kinds—one set of semi-cartilaginous cells, called pulp cells, suffer ossification; another set of cells, which are true axis cylinder nerve fibril cells, that resist ossification, and occupy the tubuli of the dentine, ossification taking place all around each fibre, numbering, in the aggregate, millions, in a large tooth. One set of cells being made up of true nerve-fibre, wholly resist ossification, or, in other words, dentification, while the other set are more or less non-vital, and readily take on ossification. In the enamel the case is different, there being but one set of non-resistant calcigorous cells. Now, in those cases of non-developed, or imperfectly developed enamel, as described in the above cases, there must be some disturbing cause, sufficient to produce this arrestation of normal development, and necessarily caused by the disease in question. This disturbing agency I regard as an acid reaction in the enamel membrane or cells, dissolving out any lime salts that may be deposited in the enamel tissue, such as any of the acids, that contain hydrogen, which may be secreted in some way in this tissue, or in the mucus membrane immediately covering this enamel membrane.

This inherited or induced syphilis in children, whether contracted during parturition, or by nurses, or any other way, at any time previous to the development of the dental structures would, in my opinion, be sufficient cause to produce this arrestation by arresting, altogether, the enamel matrix, from want of proper germinal matter, owing to defective nutrition in the parts; or if the matrix is formed at all, fails to take on ossification, from the causes assigned above.

I know of no other satisfactory explanation, and even the one given may ultimately prove to be erroneous.

I advance the above views from my own researches and observations in this direction. The profession are deficient in data on this, as well as on many other morbid processes. I have been led to believe, from my researches, that all bone absorptions, or wasting, are the direct result of acid action. Any of the hydrogen acids are capable of removing lime salts from bones; as the bones are composed of bibasic phosphate of lime, any acid, with strong affinities, readily takes up one equivalent of the bibasic phosphate, leaving a bibasic phosphate of lime, which is an acid salt, and readily soluble in almost any liquid, and is readily washed away; the carbonate of lime being also readily decomposed by any hydrogen acid. Thus the lime is disintegrated, at the same time astoine is oxidised out same as any other low form of tissue.

There can be no other way for waste of osseous tissues. Oxygen cannot take the lime out of bones, as they are already saturated with it before uniting with the phosphoric acid and carbonic acid. In this way all ravages, including normal waste of all osseous structures, are affected. The above ideas I have long entertained, and advocated in published articles on the subject.

I saw, quite recently, another strongly-marked case of furrowed enamel, of the twelve anterior teeth, The subject, a youug woman, a Creole, a dark brunette, of low origin and culture. The twelve front teeth were deeply grooved and notched, near and at their cutting edges, very similar to the two other cases described. The countenance gave no evidence of any disease: the mouth on the contrary, gave the bad fetor, similar to the last-named case, not owing to decayed teeth. I have no doubt about her case.

I have seen many others, only less strongly marked. Such cases, even in New Orleans, are not so frequent as might be supposed.

The other day a surgeon of New Orleans showed me a genuine Hunterian chancre, to all appearances, which was caused by the blood and pus of a mortified finger, that had been crushed, of a young man, 20 years old, who, when asked, about having syphilis, denied ever having had the disease. The sore is pronounced, by a number of the ablest physicians of the place, to be a genuine chancre, having all its characteristics, which was contracted some three weeks since, showing no disposition to heal by local treatment, only by constitutional mercurialization; until the last few days, has there been any improvement. During the operation, the skin of the finger was slightly abraded, and coming in contact with the blood and pus, caused the sore in question.

Is it possible for the tissues and secretions of the human body, untainted with venereal, to become specifically poisonous, so as to inoculate itself into the healthy organism, and simulate chancre, in appearance only, or with all the attributes of the genuine disease? If not, the only conclusion left in the above case is constitutional disease, and that contrary to all the above statements, this secondary, or constitutional disease, is capable of producing the primary chancre, which would, in all probability, unless checked, pass through the usual stages and sequences. The soreness of the arm, rendering it almost useless, proves the fact that the disease was traveling into the system by slow and sure marches, unless checked. Subsequently, the young man acknowledged that he had been affected.

SYPHILITIC INFECTION FROM KISSING.

Dr. F. F. Maury (in the *Medical and Surgical Reporter*) says: "I have seen two cases—one now on hand—in my private practice, where a patient, with mucous patches upon the lip, kissing another, developed upon the lip of the latter a hard chancre, with its attendants, of indurated base, involvement of the submaxillary lymphatic ganglions of the affected side, and a profuse secondary manifestation of roscola maculata."

ANOTHER CASE OF FURROWED ENAMEL.

A lady, about thirty years old, apparently of sound constitution, some of the back teeth decayed. The six front teeth, above and below, are croos-grooved—two grooves in each, about midway of the teeth, with great regularity and symmetry; no notching or pitting, as generally accompany such cases. I made inquiry whether she had ever had, whilst a child, any cutaneous disease, scarlet fever or measles. She replied, "nothing of the kind," which left the inference that there must have been some taint in her system. I could not detect any sign of venereal taint about her.

There may be other causes than constitutional taints, or any of the acute exanthemata, of furrowed enamel, that may be purely local, at a particular time, of dental germ formation during the developmental enamel processes, something like a stricture in the fibres of the investing membrane, about the time of hardening of enamel, from some oral irritation.

The ossification of enamel may be regarded a crystaline process, quite independent of the organic forces—a molecular process, according to a law of molecules, *i. e.*, polarity, the *primum mobile* of all crystaline processes.—*Nashville Jour. of Med. & Surg.*

